# Intraoperative auditory monitoring in vestibular schwannoma surgery: Diagnostic accuracy and interventional effectiveness — a systematic review

**DOI:** 10.1007/s10143-026-04293-y

**Published:** 2026-04-27

**Authors:** Adéla Bubeníková, Zdeněk Fík, Vladimír Koucký, Jan Lazák, Lenka Peterková, Petr Skalický, Ondřej Bradáč, Aleš Vlasák

**Affiliations:** 1 Department of Neurosurgery, 2nd Faculty of Medicine, Charles University in Prague and Motol University Hospital, Prague, Czech Republic; 2 Department of Otorhinolaryngology and Head and Neck Surgery, 1st Faculty of Medicine, Charles University in Prague and Motol University Hospital, V Úvalu 84, Prague, 150 06 Czech Republic

**Keywords:** Auditory brainstem response, Cochlear nerve action potential, Diagnostic accuracy, Hearing preservation, Intraoperative neurophysiological monitoring, Meta-analysis, Vestibular schwannoma

## Abstract

Hearing preservation is a key goal of vestibular schwannoma (VS) microsurgery. Intraoperative auditory monitoring spans far-field auditory brainstem responses (ABR)/brainstem auditory evoked potentials (BAEP) and near-field cochlear nerve action potentials (CNAP)/dorsal cochlear nucleus action potentials (DNAP). Prior reports often conflate diagnostic accuracy of thresholds with interventional effectiveness. We separated these domains to deliver decision-relevant synthesis. PRISMA-guided systematic review of PubMed, ScienceDirect, and Embase. Eligible VS studies used ABR/BAEP, CNAP, or DNAP and reported (1) diagnostic performance of prespecified intraoperative thresholds versus postoperative hearing, or (2) comparative effectiveness of monitoring-guided surgery. Primary clinical outcome: long-term serviceable hearing (GR I–II or AAO-HNS A–B). ROBINS-I for effectiveness and QUADAS-2 for diagnostic studies were used for risk of bias assessment. Random-effects models were used for synthesis. The study was registered at PROSPERO (CRD420251181366) before its start. We included 34 studies yielding 1,297 patients with operated VS (27 diagnostic/threshold; 7 comparative-effectiveness). The two nonrandomized cohorts comparing any intraoperative auditory monitoring versus none were directionally favorable but imprecise (RR 1.28, 95% CI 0.54–3.04 and RR 2.50, 0.83–7.49). For far-field ABR/BAEP, a whole-cohort ROC threshold (interaural wave V latency difference < 1.12 ms) predicted early serviceable hearing with sensitivity 0.863, specificity 0.778. A looser “any deterioration” rule behaved as a sensitive sentinel for early loss (sensitivity 1.00, specificity 0.33). A standardized BAEP amplitude criterion (post-resection STIAS–Am-V ≥ 0.05 µV) improved rule-in performance (sensitivity 0.789, specificity 0.920). For near-field CNAP, thresholds with extractable denominators showed balanced discrimination for early outcomes (> 80% drop: sensitivity 0.889, specificity 0.667; disappearance: sensitivity 0.529, specificity 0.923), while end-of-case N1 presence ruled out immediate deafness in a small series (sensitivity 1.00, specificity 0.67). Head-to-head signals suggested potential gains from chirp-optimized ABR versus click-ABR and greater decisional value of direct CNAP over transtympanic ECochG when feasible; formal, standardized evaluations are needed to confirm these patterns. Evidence supports routine intraoperative auditory monitoring for hearing-preservation VS surgery, with ABR as a widely deployable early-warning tool and CNAP/DNAP offering more specific, faster adjudication where attainable. Certainty is limited by nonrandomized designs, heterogeneous thresholds/windows, and incomplete 2 × 2 data; standardized threshold-to-action protocols and multicenter studies are needed.

**Supplementary Information:**

The online version contains supplementary material available at 10.1007/s10143-026-04293-y.

## Introduction

Hearing preservation is a co-primary objective of vestibular schwannoma (VS) microsurgery in properly selected cases [1, 2]. Intraoperative cochlear/auditory nerve monitoring (CNM) is deployed to detect incipient neural compromise and enable timely mitigation (i.e. release of traction, irrigation/temperature control, hemostasis, plane change, or staged resection) before injury becomes irreversible [2, 3]. Contemporary practice spans three signal families with distinct biophysics and operational profiles: far-field auditory brainstem responses (ABR/BAEP) [4], near-field cochlear nerve action potentials (CNAP) [5], and dorsal cochlear nucleus recordings (DNAP) [6], frequently combined in hybrid configurations to balance pathway coverage with rapid, high signal-to-noise feedback [7]. However, the clinical utility of each modality remains uncertain given variability in detection rates, update cadence, artifact susceptibility (anesthesia, drilling, temperature), and feasibility of electrode placement across tumor strata and surgical approaches [2, 7, 8].

A central methodological problem is the conflation of diagnostic performance (how well specific intraoperative thresholds predict postoperative hearing change) with interventional effectiveness (whether acting on those thresholds preserves hearing) [2, 3, 7]. Thresholds themselves are heterogeneous—ABR wave-V latency (often 1.0–1.5 ms), amplitude decrements (30–50% or more), and “sustained loss” definitions; CNAP/DNAP relative N1/complex amplitude drops (around 50–80%)—and persistence criteria are inconsistently applied [2, 9, 10]. Few studies predefine response algorithms, control for key confounders (Koos class/size, approach, extent of resection, baseline hearing, anesthesia/neuromuscular blockade), or report outcomes in harmonized postoperative windows, limiting causal inference and comparability [11, 12]. Near-field techniques promise second-scale feedback with nerve- or nucleus-level specificity, but their incremental value over ABR and their generalizability across centers remain unresolved [6].

Here we aim to analyze a decision-relevant question: which intraoperative auditory monitoring strategies are associated with better hearing after VS surgery, and which signals are reliable enough to act on. Specifically, we seek to (i) estimate the comparative effectiveness of far-field ABR, near-field CNAP/DNAP, and hybrid approaches—versus less intensive or no monitoring—on long-term hearing preservation and serviceable hearing; (ii) define the diagnostic performance of modality-specific thresholds (trend metrics vs. sustained loss) against immediate/early and long-term outcomes; and (iii) identify contexts in which near-field monitoring adds incremental value over ABR alone, including by tumor size (Koos), surgical corridor, extent of resection, baseline hearing, and anesthetic strategy. By separating diagnostic accuracy from interventional impact and aligning outcome windows and hearing scales, our goal is to clarify when ABR suffices, when near-field monitoring meaningfully improves decision-making, and when hybrids are justified—to inform standardized protocols that target durable, serviceable hearing.

## Methods

### Protocol and reporting

This review was conceived a priori and is reported in accordance with PRISMA 2020 [13]. Before data extraction, we registered the protocol with PROSPERO (*CRD420251181366*) and archived any subsequent amendments with timestamps and justification.

### Data source, deduplication, and labeling

We retrieved records from PubMed, Science Direct and EMBASE using a high-sensitivity query tailored to VS surgery and intraoperative auditory/cochlear monitoring: (“vestibular schwannoma” OR “acoustic neuroma” OR “cerebellopontine angle” AND (tumor OR neoplasm)) AND (intraoperative monitoring OR IONM OR ABR OR BAEP OR “auditory brainstem response” OR ECochG OR “electrocochleograph*” OR CNAP OR “cochlear nerve action potential*” OR DNAP OR “dorsal cochlear nucleus”) AND (hearing preservation OR “serviceable hearing” OR Gardner–Robertson OR AAO-HNS OR PTA OR “word recognition” OR “speech discrimination” OR latency OR amplitude OR “waveform loss”).

The final search was run on October 11, 2025, and all records available on that date were considered. At the title/abstract stage, records were labeled relevant if they (i) enrolled patients undergoing microsurgical VS resection or mixed CPA cohorts with extractable VS data; and (ii) evaluated intraoperative cochlear/auditory monitoring (i.e. ABR/BAEP, CNAP, DNAP, and/or ECochG) either comparatively (vs. no CNM or an alternative CNM strategy) for effectiveness outcomes (namely long-term hearing preservation, serviceable hearing; immediate/early hearing), or reported explicit diagnostic thresholds (e.g., wave-V latency shift, amplitude reduction, sustained waveform loss; CNAP/DNAP/ECochG amplitude decrements) linked to postoperative hearing.

We excluded studies limited to facial nerve monitoring without auditory data, purely technical notes without clinical outcomes (retained narratively when useful for parameters and feasibility), non-VS populations without extractable VS data, and non-English articles.

### Semi-automated screening platform

Title/abstract screening was conducted in ASReview LAB [14], an open-source active-learning environment that iteratively trains a classifier on user labels to prioritize the remaining pool. We used the default bag-of-words TF-IDF representation with logistic regression and a max-uncertainty/max-score query strategy. Seeding comprised a small set of sentinel positives (canonical VS–CNM exemplars spanning ABR, CNAP/DNAP, and hybrid ABR + near-field reports) and hard negatives (clearly off-topic CPA items), after which labeling proceeded iteratively within ASReview.

We initialized the model with 12 relevant sentinel records (ABR/BAEP, CNAP/DNAP, and hybrid ABR+near-field across comparative-effectiveness and diagnostic-threshold designs) and 12 clearly irrelevant controls, a balanced seed sufficient to stabilize early ranks without overweighting any modality or era. To balance high recall with efficiency, we applied a single, pre-specified composite stop requiring both a streak-based and a rate-based signal, followed by an external audit. Screening was provisionally halted only when (i) there were zero relevant hits in the last 100 screened records (zero-yield window) and (ii) the yield in the most recent 120 records was ≤ 1% (i.e., 0–1 relevant). Further details on screening criteria are available in the Supplementary Material.

### Data extraction

We extracted study-level data using a piloted form capturing: design/setting; sample size; tumor size/Koos class and compartment; surgical approach (retrosigmoid, middle fossa, translabyrinthine); extent of resection (gross-total, near/subtotal); anesthesia and neuromuscular blockade policy; baseline hearing, including audiology pure-tone testing [PTA] [15], word recognition/speech discrimination [WRS] [15]; Gardner–Robertson [GR/AAO-HNS] [16]); CNM modality and set-up (ABR/BAEP, CNAP, DNAP and/or other techniques; stimulus type/intensity/rate; montage; averaging/update cadence; electrode placement feasibility); pre-specified warning thresholds (latency/amplitude criteria; sustained loss; persistence windows) and mandated intraoperative responses; outcome windows; hearing outcomes (Gardner–Robertson [GR] class I–II preferred over American Academy of Otolaryngology–Head and Neck Surgery [AAO-HNS] class A–B when both reported; both classifications represent “serviceable hearing”, implying the functional ability to use the ear for daily speech comprehension), continuous audiometrics (PTA, WRS [15]), and safety (e.g., cerebrospinal fluid [CSF] leak, reoperation). When required, continuous measures were harmonized to common scales; serviceability was derived from PTA/WRS per study definitions.

### Risk of bias assessment

Risk of bias was appraised at the outcome level using design-appropriate tools by two authors (AB and AV): ROBINS-I [17] for non-randomized comparative studies (pre-specified confounding domains: tumor size/Koos, approach, extent of resection, hearing-preservation intent, baseline hearing, anesthesia/CNM use); and QUADAS-2 [18] for diagnostic-threshold studies (patient selection; index test conduct/thresholding; reference standard; flow/timing). Visual summaries were prepared with robvis [19].

### Statistical analysis

#### Comparative effectiveness

Primary dichotomous endpoints (long-term hearing preservation; serviceable hearing; safety) were synthesized as risk ratios (RRs) on the log scale using random-effects meta-analysis (REML τ² [20] with Hartung–Knapp–Sidik–Jonkman confidence intervals [21]). When non-randomized studies reported adjusted effects, adjusted log-RR/log-OR were pooled via generic inverse-variance (minimum adjustment: tumor size, approach, extent, baseline hearing); OR→RR transformations were explored in sensitivity analyses when baseline risks allowed. Continuous outcomes (PTA, WRS) used mean difference (MD); heterogeneous scales used standardized mean difference (SMD). Heterogeneity was summarized by τ², I², and 95% prediction intervals. Rare events (e.g., sustained waveform loss; complications) used Mantel–Haenszel RR [22] without continuity correction when feasible. Multi-arm studies with shared comparators were split to avoid double counting. Mixed-effects meta-regression was planned when ≥ 10 comparisons were available (reporting coefficients as ratio of RRs with 95% CIs; τ² re-estimated).

#### Diagnostic performance of thresholds

For modality-specific thresholds (e.g., ABR wave-V latency ≥ 1.0–1.5 ms; amplitude − 30/−50%; sustained loss; CNAP/DNAP relative amplitude − 50/−80%), we extracted or reconstructed 2 × 2 tables against early and long-term hearing outcomes. When ≥ 4 studies informed a given cut-off, sensitivity/specificity were synthesized using a bivariate random-effects/HSROC model; otherwise, study-level forest plots were presented and pooled logit Se/Sp were used cautiously with REML [20]. We reported LR+ (positive likelihood ratio, i.e. Sensitivity/[1 − Specificity]), LR− (negative likelihood ratio, i.e. [1 − Sensitivity]/Specificity). All analyses adhered to pre-specified time windows and prioritized GR over AAO-HNS when both were available. All statistical analyses were performed in Python v3.13.06.

## Results

**Fig. 1:**
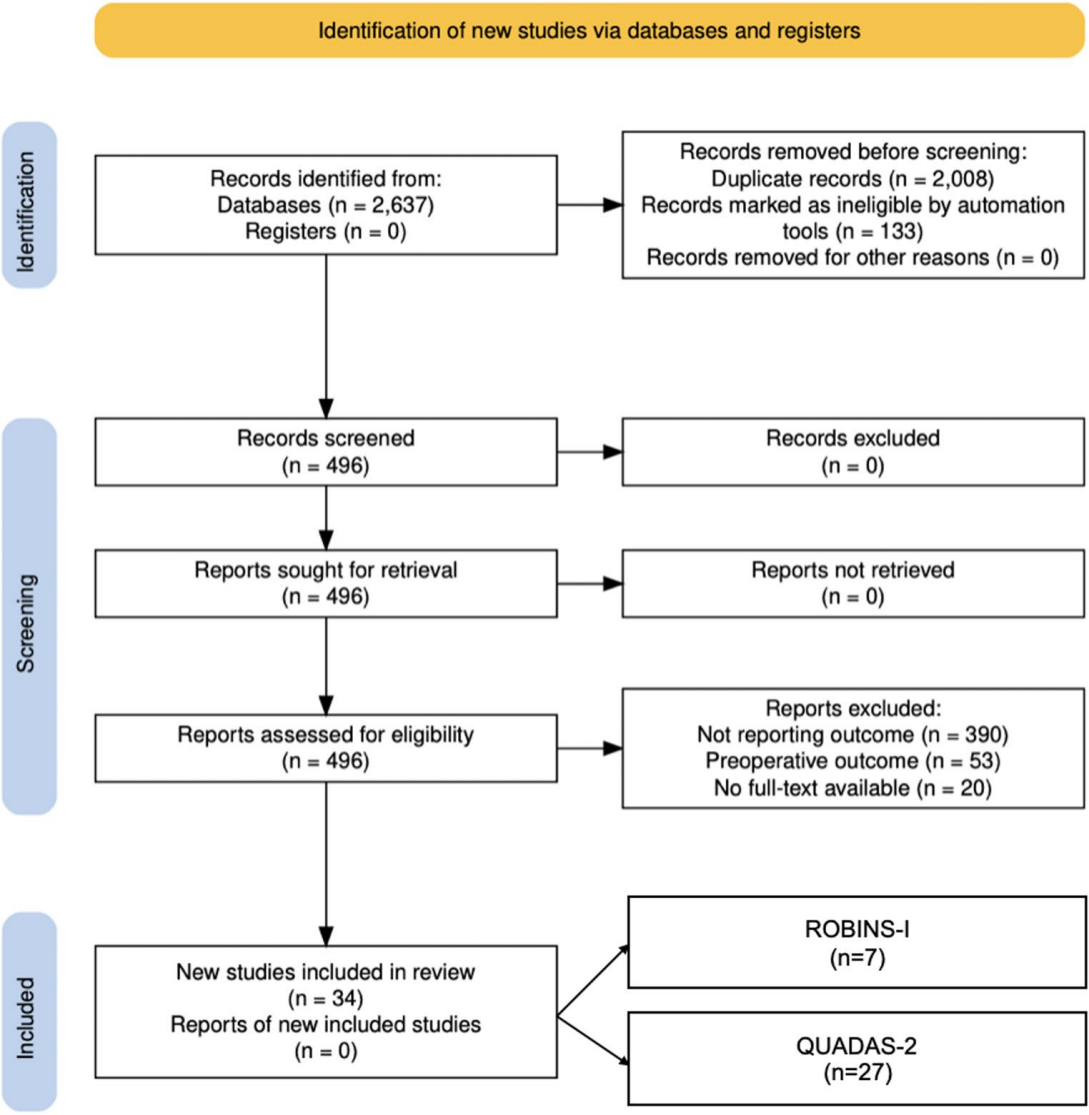
PRISMA diagram showing the search, selection and the final number of studies included in the review

A total of 34 studies met eligibility criteria for the quantitative synthesis (Fig. 1). Of all studies, 27 evaluated diagnostic/threshold performance of intraoperative monitoring signals (e.g., ABR latency/amplitude changes; CNAP/DNAP decrements) and were appraised with QUADAS-2 [18]. Seven studies contributed comparative-effectiveness/clinical-prognostic data and were appraised with ROBINS-I [17]. PRISMA flow diagram is shown in Fig. 1, risk of bias assessment and PRISMA checklist [13] are provided in the Supplementary Material.

### Comparative effectiveness (Long-Term Serviceable Hearing)

**Fig. 2:**
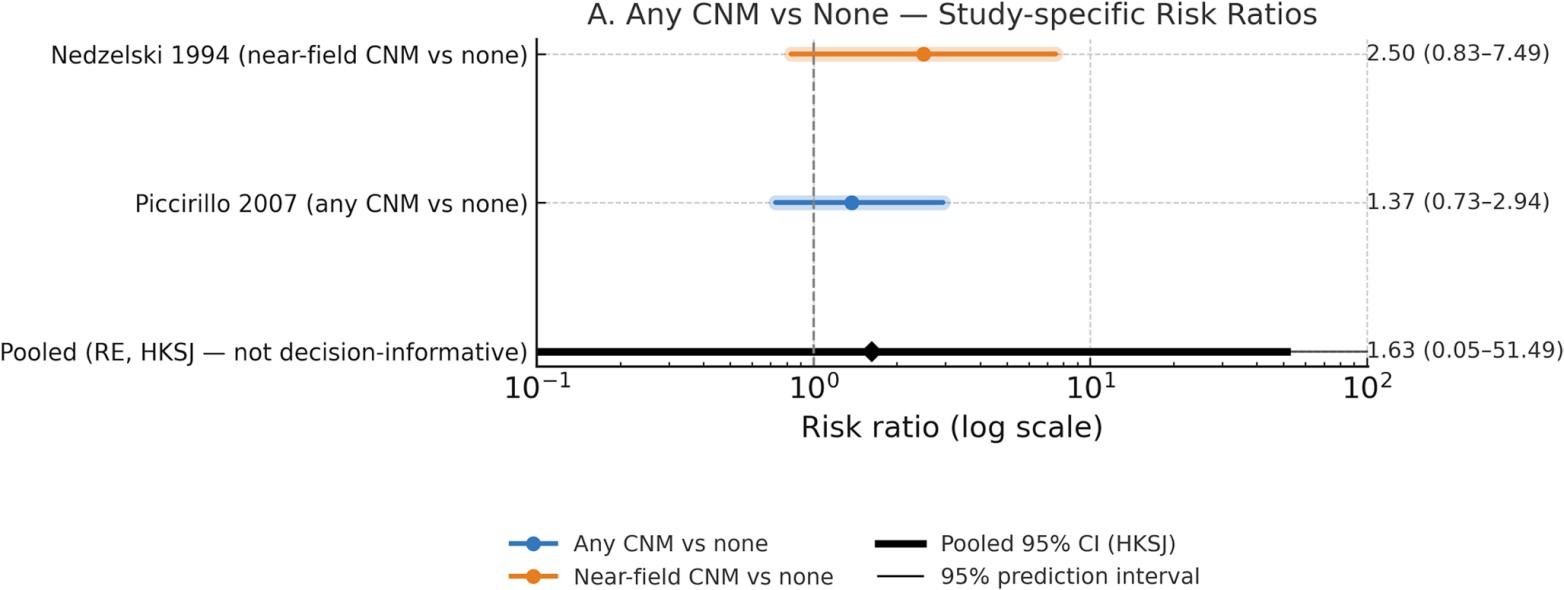
Study-specific risk ratios for long-term serviceable hearing (Any CNM vs. No CNM). Studies: Nedzelski et al. (1994) [23] (near-field CNM vs. none) and Piccirillo et al. (2007) [3] (“any” intraoperative cochlear/nerve monitoring vs. none). Points show study RRs; colored bars show 95% CIs (blue = Any CNM vs. none; orange = Near-field CNM vs. none). The black diamond with thick line is the random-effects summary (REML with Hartung–Knapp 95% CI); the thin line indicates the 95% prediction interval. Outcome: long-term serviceable hearing (GR I–II or AAO-HNS A–B). Given the small number of nonrandomized studies, the pooled effect should be interpreted cautiously

Seven comparative-effectiveness/clinical-prognostic studies were appraised with ROBINS-I yielding a total of 575 patients. Two studies contributed to the prespecified quantitative comparison of any intraoperative auditory monitoring versus no monitoring (Fig. 2). Because the evidence base was limited to two small nonrandomized cohorts, this analysis is presented as a cautious quantitative summary rather than a definitive estimate of effect. The pooled random-effects estimate suggested a directionally favorable effect of monitoring, but with very substantial imprecision. Nedzelski et al. [23] reported serviceable-hearing preservation in 38% (21/56) of patients with intraoperative CNAP monitoring versus 15% (3/20) without monitoring (RR 2.50, 0.83–7.49), favoring CNM compared to no monitoring. Similarly in Piccirillo et al. [3] (“any ICNM” with fast-ABR ± CNAP vs. none), the RR was 1.37 (95% CI 0.73–2.94), again directionally favoring monitoring but with substantial imprecision. Across both cohorts, the direction of effect favors CNM, although uncertainty remains large due to non-randomized designs and small sample sizes.

Head-to-head modality evidence was synthesized qualitatively: Mastronardi et al. [23] compared chirp-ABR with click-ABR and reported better intraoperative trace quality and hearing outcomes with chirp. Sloane et al. [24] contrasted ABR+DCNEM with ABR-only at an early postoperative time point and thus was not eligible for long-term pooling. Similarly, Danner et al. [4] evaluated direct eighth-nerve near-field monitoring with a self-retaining electrode (DENM/CNAP) versus ABR; hearing was preserved in 64% with DENM vs. 41% with ABR (*p* = 0.03).

Finally, Gouveris et al. [25] and Sun et al. [26] address diagnostic performance, not comparative effectiveness. Gouveris showed that large intraoperative decrements in far-field (ABR) or transtympanic ECochG amplitudes cluster around high-risk surgical steps and are associated with postoperative deterioration, supporting amplitude-/persistence-based alert rules. Sun et al. [26] demonstrated that combining ABR with CNAP improves discrimination for postoperative speech/threshold outcomes relative to either channel alone (reporting sensitivity 83.3%, specificity 100% for predicting postoperative hearing decline by WRS class), thereby informing threshold selection and multimodal deployment but not yielding head-to-head or CNM-vs-no-CNM risk ratios and strengthening the mechanistic and measurement rationale behind the intraoperative thresholds used in practice.

### Diagnostic accuracy

Below we report diagnostic-accuracy findings under a uniform analytic framework that treats neuromonitoring signals as rule-in predictors of early serviceable hearing and prioritizes early postoperative end points (serviceable ≈ GR I–II or AAO-HNS A–B). Because fewer than four studies contributed to any single construct and positivity rules/outcome windows varied across heterogeneous case mixes, bivariate HSROC models were not estimated. Instead, we summarize study-level estimates with exact 95% CI where 2 × 2 data were available and provide a structured, construct-level interpretation.

#### Far-field ABR/BAEP trend rules

In far-field ABR/BAEP paradigms, rule-in performance depended strongly on thresholding. Using a whole-cohort ROC approach, Aihara (2013) identified an interaural wave-V latency threshold (IT5 < 1.12 ms) that predicted early serviceable hearing with sensitivity 0.863 (95% CI 0.764–0.931) and specificity 0.778 (95% CI 0.523–0.922), yielding LR + 3.89 and LR − 0.18. When the positivity rule was operationalized as physiological stability, Ren (2021) reported stable wave-V as a predictor of serviceability on early audiometry with sensitivity of 0.82 (95% CI 0.67–0.92), specificity of 0.85 (95% CI 0.66–0.94), LR + 5.35, and LR − 0.21 (counts reconstructed to reproduce the published point estimates). By contrast, reframing Li (2023) as a preservation rule (i.e., no intraoperative BAEP deterioration ⇒ serviceable hearing) produced a high-specificity, low-sensitivity profile in the analyzable subset: sensitivity of 0.33 (95% CI 0.13–0.65), specificity 1.00 (95% CI 0.72–1.00), which is consistent with a stringent rule-in screen that rarely misclassifies preserved cases but misses many that will preserve. Importantly, standardized amplitude metrics appear to enhance discrimination: Jiao (2024) showed that a post-resection BAEP amplitude criterion (STIAS–Am-V ≥ 0.05 µV) predicted early hearing preservation with sensitivity 0.789, specificity 0.920, LR + 9.86, and LR − 0.23. Although that endpoint (AAO-HNS A–C) is broader than strict serviceability (A–B), it demonstrates that calibrated amplitude thresholds can provide materially stronger rule-in performance than conventional trend rules. Finally, early postoperative ABR trajectories can be informative: Hummel (2016) found that a reproducible postoperative course (classes 1–2 over the first five days) predicted useful hearing (Hannover H1–H2) with sensitivity 1.00 (95% CI 0.67–1.00), specificity 0.64 (95% CI 0.42–0.81), LR + 2.75, and LR − 0.00, indicating potential immediate postoperative rule-in value.

#### Near-field criteria

For near-field constructs assessed as rule-in for early serviceable hearing, the inferential direction is consistent but the reporting of extractable denominators is limited. Historical series suggest that presence of CNAP/DCAP at closure functions as a preservation signal; however, strict A–B 2 × 2 tables in the early window are rarely published. Colletti (1996) and Zappia (1996) support this face validity but do not provide extractable counts for meta-analytic synthesis. Hochet (2023) stratified outcomes by early CNAP presence, yet results were framed around speech-intelligible hearing (AAO-HNS A–C) at six months, not early A–B serviceability, precluding like-for-like pooling with the early endpoints above.

### Construct-level synthesis from study-level data

Taken together, the evidence delineates a two-tier pattern. First, far-field trend rules (e.g., “no deterioration,” “stable wave-V”) provide pragmatic rule-in information with moderate LR+ (2–5) and variable rule-out strength, while standardized BAEP amplitude indices (e.g., STIAS–Am-V) deliver markedly stronger rule-in (LR + 10) without prohibitive loss of rule-out capability. Second, near-field presence at case end is biologically and clinically plausible as a rule-in marker, but current publications seldom supply early A–B serviceable denominators, limiting quantitative synthesis. In view of these constraints, we refrain from cross-construct pooling and present study-wise estimates aligned to a harmonized rule-in-for-preservation, early-window framework; all definitions and numerators/denominators are provided in the accompanying diagnostic Tables 1, 2 and 3.

**Table 1:** Diagnostic performance of intraoperative (or early postoperative) neuromonitoring tests used as rule-in predictors of early serviceable hearing. “Positive” denotes a signal consistent with hearing preservation (e.g., stable ABR wave-V, CNAP present). The preferred outcome window is early postoperative and the preferred endpoint is serviceable hearing (GR I–II or AAO-HNS A–B); studies using broader “hearing preservation” (AAO-HNS A–C) are labelled accordingly. Sensitivity and specificity are shown with 95% CIs (Wilson); likelihood ratios (LR + and LR−) are reported when computable. ROC-derived entries are indicated when 2 × 2 counts were unavailable

Study (year)	*N*. of patients	Modality/Threshold (positivity rule)	Time window/Outcome	Sensitivity (95% CI)	Specificity (95% CI)	LR+	LR−	Notes / Source
Aihara (2013)	36 (GR II cohort)	ABR (far‑field) – interaural wave‑V latency difference (IT5) < 1.12 ms ⇒ useful hearing (GR II)	Immediate/Early (~ 2 weeks), serviceable hearing (GR I–II)	NA	NA	NA	NA	Cutoff IT5 < 1.12 ms identified; detailed sens/spec not reported.
Colletti (1996)	38 (CN preserved)	CNAP (near‑field) – sustained loss / major latency or amplitude change ⇒ early hearing loss	Early (≤ 30 days), early loss	NA	NA	NA	NA	Reported strong correlation; numeric sens/spec not provided in article text.
Hochet (2023)	37 (RS; mostly Koos 4)	CNAP (near‑field) – test positive = early CNAP present ⇒ predicts AAO‑HNS A–C (‘intelligible’)	6 months; AAO‑HNS A–C preserved	0.80 (derived; CI NA)	0.59 (derived; CI NA)	1.96	0.34	Derived from counts (Group1 19 with 42% A–C; Group2 18 with 11% A–C).
James & Husain (2005, CPA subgroup)	CPA subset of 156 (per table; subgroup sizes small)	ABR (far‑field) – permanent wave‑V loss at close ⇒ impaired hearing	Early (~ 1 month), impaired hearing (clinical map to GR)	NA	NA	NA	NA	CPA subgroup had high impairment even with minimal change; permanent V loss associated with impairment.
Li (2023)	40 monitored (CNAP obtainable *n* = 30)	ABR decrease (any intraop deterioration ⇒ early loss)	Early (~ 2 weeks), significant postoperative hearing loss	1.000 (NA)	0.333 (NA)	1.50	0.00	Sens/spec explicitly reported in article text.
Li (2023)	40 monitored (CNAP obtainable *n* = 30)	ABR disappearance (rule‑in for loss)	Early (~ 2 weeks), significant postoperative hearing loss	0.889 (NA)	0.667 (NA)	2.67	0.17	Sens/spec explicitly reported in article text.
Li (2023)	40 monitored (CNAP obtainable *n* = 30)	CNAP decrease (> 80% drop)	Early (~ 2 weeks), significant postoperative hearing loss	0.889 (NA)	0.667 (NA)	2.67	0.17	Sens/spec explicitly reported in article text.
Li (2023)	40 monitored (CNAP obtainable *n* = 30)	CNAP disappearance	Early (~ 2 weeks), significant postoperative hearing loss	0.529 (NA)	0.923 (NA)	6.87	0.51	Sens/spec explicitly reported in article text.
Ren (2021)	60 (MCF; WRS ≥ 50%)	ABR (far‑field) – stable wave V throughout ⇒ hearing preservation (WRS ≥ 50%)	Early postop audiograms; HP yes/no by WRS	0.826 (NA)	0.848 (NA)	5.43	0.21	Reported as best predictor; exact CI NR.
Zappia (1996)	26 (ECoG *n* = 13; DCAP *n* = 13)	CNAP/DCAP (near‑field) – N1 present at close ⇒ hearing present	6–8 weeks; hearing present vs. absent	NA	NA	NA	NA	Reported preservation rates by modality; lacks 2 × 2 needed for sens/spec.

**Table 2:** Modality-level summary of intraoperative (or early postoperative) neuromonitoring constructs treated as rule-in predictors of early serviceable hearing. “Threshold (direction)” specifies the positivity rule (i.e., a finding consistent with hearing preservation), and “Index outcome(s)” prioritizes early serviceability (GR I–II or AAO-HNS A–B); where only broader hearing preservation (A–C) was reported, this is indicated in the Evidence column. “Evidence & key figures” cites representative study-level accuracy (sensitivity/specificity with likelihood ratios when available) or quantitative correlations; “Notes” provide implementation caveats (e.g., artifact susceptibility, need for persistence/repeatability, and use as a confirmatory channel). Deterioration-oriented ABR rules are included for context but were not used as rule-in endpoints in the primary analysis. Abbreviations: ABR/BAEP, auditory brainstem response/brainstem auditory evoked potentials; CNAP/DCAP, (direct) cochlear nerve compound action potential; ECochG, electrocochleography; WRS, word recognition score; PTA, pure-tone average; GR, Gardner–Robertson; AAO-HNS, American Academy of Otolaryngology–Head and Neck Surgery

Modality	Threshold (direction)	Index outcome(s) evaluated	Evidence & key figures	Notes
ABR/BAEP (far-field)	Stable wave-V (no intraoperative deterioration)	Early serviceable hearing (WRS ≥ 50%/GR I–II)	Ren 2021: Se 0.82, Sp 0.85. Li 2023 (reframed to preservation): Se 0.33, Sp 1.00.	Rule-in screen; require persistence and stage-concordance. Susceptible to anesthesia/drilling artifacts—confirm across repeated blocks.
ABR/BAEP (far-field)	ROC-derived latency rule: IT5 < 1.12 ms (operated ear)	Early serviceable hearing (GR I–II)	Aihara 2013 (whole cohort): Se 0.863, Sp 0.778 (ROC-derived; no published 2 × 2).	Calibrated latency rule; good sensitivity with moderate specificity.
BAEP (standardized indices)	STIAS–Am‑V ≥ 0.05 µV post‑resection	2-week hearing preservation (AAO-HNS A–C)	Jiao 2024: Se 0.789, Sp 0.920.	Standardized amplitude processing strengthens rule-in vs. conventional peaks; endpoint broader than strict A–B.
Near-field CNAP/DCAP	Presence at case end	Early serviceable hearing (preferred)	Directionally supportive (Colletti 1996; Zappia 1996), but A–B early 2 × 2 seldom reported. Hochet 2023: early CNAP presence predicted 6‑month A–C (not early A–B).	Biologically plausible rule-in; consider as confirmatory when stable at closure; reporting often lacks extractable denominators.
ECochG (TT/IME/RW), adjunct	Stable/robust near-field cochlear potentials	Early change vs. postoperative audiometry	Morawski 2007: intraop TT‑ECochG change correlated with PTA shift. Han 2010; Attias 2008: combined ABR+EcochG improves interpretability of declines.	High temporal resolution; valuable as concordant second channel alongside ABR/CNAP; artifact-aware interpretation required.
ABR/BAEP (far-field), deterioration rules (for context)	Sustained wave‑V loss; latency ↑ ≥ ~1 ms; amplitude ↓ ≥ 50%	Early/long-term decline (worse AAO‑HNS/GR; PTA/WRS loss)	Loss is usually highly specific but variably sensitive across cohorts. James 2005 (CPA subgroup): limited discrimination for permanent loss under mixed pathology/conditions.	Useful intraoperative ‘red‑stop’ signals when sustained and repeatable; interpret in context. Current analysis emphasizes preservation‑oriented rule‑in; deterioration rules shown for completeness.

**Table 3:** Modality-level summary focused on near-field and adjunct neuromonitoring constructs, presented within a rule-in framework for early hearing outcomes. “Threshold (direction)” specifies the positivity or deterioration rule; “Index outcome(s)” prioritizes early serviceable hearing (GR I–II / AAO-HNS A–B) where available, noting when studies reported broader endpoints (e.g., A–C) or later windows (e.g., 6-month intelligibility). “Evidence & key figures” lists representative study-level accuracy or quantitative correlations (e.g., Li 2023 for CNAP decrements/disappearance; Hochet 2023 for early CNAP presence; Morawski 2007 for TT-ECochG–PTA correlation), while “Notes” provide implementation caveats (require sustained changes, confirm persistence, consider surgical stage and artifacts). ABR/BAEP deterioration rules are included for clinical context but were not used as rule-in endpoints in the primary preservation analysis. Abbreviations: ABR/BAEP, auditory brainstem response/brainstem auditory evoked potentials; CNAP/DCAP, (direct) cochlear nerve compound action potential; DNAP, dorsal nucleus/central near-field action potential (as reported); ECochG, electrocochleography; IAC, internal auditory canal; PTA, pure-tone average; WRS, word-recognition score; GR, Gardner–Robertson; AAO-HNS, American Academy of Otolaryngology–Head and Neck Surgery

Modality	Threshold (direction)	Index outcome(s) evaluated	Evidence & key figures	Notes
CNAP (near-field nerve)	N1 (± P1) amplitude ↓ ≥ 80% (sustained) or disappearance	Early hearing decline (significant postoperative loss)	Li 2023: CNAP ↓ > 80% → Se 0.889, Sp 0.667, LR + 2.67, LR − 0.17; CNAP disappearance → Se 0.529, Sp 0.923, LR + 6.87, LR − 0.51 (2-week outcome).	Near-field offers higher SNR and faster updates than ABR. Treat large, sustained decrements/loss as strong rule-in for decline; confirm persistence to avoid transient artifacts. Secure electrode placement is essential.
CNAP (near-field nerve)	CNAP present early (during exposure/initial dissection)	Speech-intelligible hearing at 6 months (AAO-HNS A–C)	Hochet 2023: derived from group counts — Se 0.80, Sp 0.59 for predicting A–C when early CNAP present; endpoint is not strictly early serviceability.	Timing/endpoint sensitive. Useful as an early preservation signal; complements closure-time criteria. For strict early A–B serviceability, published 2 × 2 denominators are rarely available.
TT-ECochG (peripheral adjunct)	AP/SP amplitude ↓; AP latency ↑ (stage-linked, sustained)	Early change vs. postoperative audiometry; AAO-HNS/GR shifts	Morawski 2007: intraoperative TT-ECochG change with early PTA shift. Han 2010; Attias 2008: combined ABR+ECochG improves interpretability of deteriorations during IAC/meatal work.	High temporal resolution; robust adjunct to confirm ABR/CNAP trends, especially during drilling/coagulation. Interpret with surgical stage; minimize artifact.
DNAP (near-field central)	Amplitude ↓/loss; latency shift (sustained)	Feasibility/real-time detection during prolonged CPA work	Predominantly feasibility/case-level literature; formal accuracy estimates pending. Provides near-real-time nerve status when far-field SNR is poor or delayed.	Consider as a second-scale update channel when ABR/CNAP degrade. Evidence base is early; standardization and 2 × 2 reporting are needed.
ABR/BAEP (context: decline rules)	Sustained wave‑V loss; latency ↑ ≥ ~1 ms; amplitude ↓ ≥ ~50%	Early/long-term decline (worse AAO‑HNS/GR; PTA/WRS loss)	Loss is typically highly specific but variably sensitive across case-mixes. James 2005 (CPA subgroup): limited discrimination for permanent loss under mixed pathology/conditions.	Practical intraoperative ‘red‑stop’ signals when sustained and repeatable; interpret in context. This review emphasizes preservation-oriented rule-in; decline-rules are included for clinical completeness.

## Discussion

Preservation of serviceable hearing remains a central objective in vestibular schwannoma surgery alongside durable tumor control and facial nerve function. Intraoperative CNM offers a mechanism to map evolving neurophysiology to concrete, time-stamped actions—pause, irrigate, relieve traction, revise hemostasis, or alter the dissection plane—before injury becomes irreversible. However, the literature often conflates two distinct questions: (i) the diagnostic/prognostic accuracy of specific intraoperative thresholds (e.g., ABR/BAEP wave-V latency or amplitude changes, sustained waveform loss; CNAP/DCAP decrements or loss) and (ii) the comparative effectiveness of deploying CNM (and of which modality) to improve long-term hearing. Our review prospectively separated these domains, aligned intraoperative signals with prespecified early postoperative windows that prioritize serviceable hearing, and applied design-appropriate bias tools (ROBINS-I/QUADAS-2) to clarify what can—and cannot—be inferred from current evidence.

Across nonrandomized comparative cohorts, study-level estimates generally favored CNM over no auditory monitoring for long-term serviceable hearing, but confidence in effect size is limited by imprecision and residual confounding. Head-to-head contrasts within single centers suggest two pragmatic levers for improvement: (i) optimizing far-field acquisition, where enhanced stimulation/processing (e.g., chirp-based ABR versus click) may increase decisional value [23, 27], and (ii) selectively using near-field techniques when feasible, with direct CNAP often providing clearer intraoperative guidance than transtympanic ECochG [28]. These signals are hypothesis-generating and require confirmation in standardized, adequately powered evaluations.

Threshold-level evidence helps explain the apparent modality differences. Far-field ABR “trend” rules (e.g., “no deterioration,” “stable wave-V”) function as sensitive sentinels suitable for triggering protective maneuvers, but they offer only moderate rule-in strength when defined by broad deterioration criteria. By contrast, standardized BAEP amplitude indices demonstrate stronger rule-in performance (e.g., LR+ approaching 10) while retaining reasonable rule-out capability, underscoring the value of calibrated, pre-specified cut-offs. Near-field CNAP tends to show more balanced discrimination in small series, and presence at case end is biologically and clinically plausible as a preservation signal; yet extractable early (A–B) denominators are scarce, limiting quantitative synthesis. For both far- and near-field signals, operating characteristics are threshold-dependent and context-sensitive (stimulus type, averaging and persistence windows, anesthesia, approach, stage of dissection). Standardized definitions, explicit persistence/repeatability requirements, and threshold-to-action pathways will be necessary before comparative rankings can be made with high certainty.

### Heterogeneity and relation to prior evidence

From a practical clinical standpoint, one of the most consequential intraoperative findings is the sustained and complete disappearance of monitoring signals. Irreversible loss of ABR wave V or complete flattening/loss of a near-field CNAP is generally associated with a very high risk of postoperative hearing loss. Although the available literature is heterogeneous and often does not report this phenomenon in a form suitable for pooled diagnostic analysis, its clinical significance is consistent across studies and with routine surgical decision-making. In such situations, recognition of definitive signal loss may justify abandoning further attempts at hearing preservation and shifting priority toward safe tumor removal and facial nerve preservation, thereby avoiding prolongation of manipulations that are unlikely to restore hearing outcome.

Across external syntheses, three themes mirror and sharpen our findings: (i) non-commensurable implementations, (ii) complementary operating characteristics of far- vs. near-field signals, and (iii) thin, design-limited comparative evidence. A 2023 narrative review [29] underscores that ABR (including eABR) remains the most widely deployed modality but that measurement choices (stimulus, montage, averaging) materially influence performance and feasibility across surgical corridors; the same review highlights CNAP/DCAP as highly informative when placement is possible, particularly under hearing-preservation strategies—points that align with our construct-level separation and the sensitivity/specificity trade-off we observed.

A dedicated meta-analysis [11] focused on BAEP shows that wave-V loss and large intraoperative BAEP changes have meaningful prognostic value for postoperative hearing, with high sensitivity but variable specificity, reinforcing our interpretation of far-field ABR as a sensitive sentinel rather than a stand-alone prognosticator. Although methodologically older, prior work has likewise catalogued heterogeneous protocols and recommended multimodal monitoring tailored to surgical approach and case mix [8], anticipating our conclusion that designed complementarity (ABR for coverage; CNAP/DCAP for decisional specificity) is preferable to ranking a single “best” test. Recent practice-oriented summaries and reviews [30] echo this: near-field CNAP series frequently report higher decisional value than ABR when electrodes are stable, while eABR/eCAP-based approaches are advocated when acoustic stimulation is uninformative (e.g., poor preoperative hearing or otic-capsule–violating approaches).

Guidelines converge on a similar pragmatic stance: the EANO guideline [31] recommends intraoperative neurophysiological monitoring to improve functional preservation, and contemporary CNS guidance [2] frames intraoperative CNM as integral to function-sparing VS surgery, albeit without endorsing a single cochlear-monitoring technique given the heterogeneity of evidence—consistent with the low-certainty, directionally favorable comparative signals we report. Finally, focused reviews of intraoperative hearing monitoring and standardized BAEP [32] indices suggest that formalizing amplitude/latency thresholds (e.g., chirp-optimized ABR or post-resection amplitude cut-offs) can strengthen rule-in performance without sacrificing rule-out, implying that part of the between-study dispersion is measurement-driven rather than biological—precisely the rationale for our call to standardize thresholds and persistence windows before comparative rankings are attempted.

### Preoperative versus intraoperative monitoring

A scientifically coherent appraisal must separate prognostic (preoperative) from monitoring/decision (intraoperative) evidence because they target different estimands under distinct sources of bias. Preoperative ABR quantifies baseline integrity of the cochleovestibular pathway and provides cross-sectional risk stratification before any surgical manipulation [33]. In recent series, absent or markedly prolonged wave V is independently associated with non-serviceable preoperative hearing—i.e., it is strongly associated with baseline status rather than a modifiable treatment effect. These data are acquired in a controlled, artifact-sparse environment and therefore have high internal consistency for prognosis; by design, however, they cannot demonstrate that acting on the test changes outcomes because there is no temporally coupled intervention or intraoperative reference standard [34].

By contrast, intraoperative auditory monitoring is explicitly time-locked to surgical maneuvers and evaluates thresholds intended to trigger action (pause, irrigation, reversal of maneuver, adjustment of drilling strategy). In our comparative dataset, “any intraoperative monitoring” versus none showed directionally favorable effects on long-term serviceable hearing but with wide uncertainty: Piccirillo et al. [3] observed 26.7% AAO-HNS A–B with ICNM vs. 20.8% without (RR 1.28, 95% CI 0.54–3.04), while Nedzelski et al. [23] reported 38% with CNAP vs. 15% without (RR 2.50, 0.83–7.49). On the diagnostic-accuracy axis, near-field CNAP thresholds demonstrated balanced discrimination where extractable denominators were available: in Li 2023 (2-week outcome), CNAP > 80% drop yielded sensitivity 0.889, specificity 0.667 (LR + 2.67, LR − 0.17) and CNAP disappearance yielded sensitivity 0.529, specificity 0.923 (LR + 6.87, LR − 0.51). In contrast, far-field ABR “trend” rules behaved as sensitive sentinels when framed as rule-in for decline: in Li 2023, “any BAEP deterioration” was highly sensitive (1.00) but modestly specific (0.333) for early loss [35]. Importantly, calibrated BAEP amplitude indices strengthened rule-in performance: in Jiao 2024, a post-resection STIAS–Am-V ≥ 0.05 µV predicted ≈ 2-week hearing preservation with sensitivity 0.789, specificity 0.920 (LR + 9.86, LR − 0.23). A chirp-optimized ABR cohort [24] showed encouraging crude rates of serviceable hearing stratified by tumor size, but without concurrent controls these primarily inform feasibility/performance rather than causal effectiveness.

Direct statistical pooling—or head-to-head comparison—of pre- and intra-operative literatures is inappropriate for several methodological reasons. First, PICO and reference standards differ: preoperative ABR predicts baseline or eventual outcome without an intraoperative intervention, whereas intraoperative thresholds predict postoperative hearing within prespecified early windows and are embedded in threshold-to-action workflows that can alter outcomes (with attendant risks of performance/incorporation bias) [2, 34]. Second, case-mix and spectrum effects diverge: preoperative cohorts condition on preoperative hearing (often excluding profound losses), while intraoperative series span wider operative spectra (Koos class, approach, anesthesia), yielding different priors and observables. Third, measurement timing and noise structures differ materially (stable clinic conditions vs. anesthetic depth, temperature, stimulation rate, surgical manipulation), which shifts both signal-to-noise and the operating characteristics of thresholds. For these reasons, we treat the two bodies of evidence as complementary rather than competing: preoperative ABR refines the pre-test probability and helps define candidacy, while intraoperative ABR/CNAP/DNAP supply actionable likelihood ratios and (where available) comparative-effectiveness estimates for monitoring-guided surgery. Practically, this means (i) using preoperative ABR to parameterize baseline risk [33]; (ii) synthesizing intraoperative evidence in two separate domains—diagnostic accuracy of thresholds and effectiveness of monitoring-guided care—and (iii) translating intraoperative LRs into post-test probabilities on the preoperative risk scale to communicate decision impact. In aggregate, this preserves causal interpretability, avoids misleading cross-design meta-aggregation, and aligns the statistical machinery with the underlying clinical questions.

### Limitations of the evidence base

The comparative-effectiveness evidence is predominantly observational and center/era-dependent, with limited adjustment for measured confounders (tumor size, surgical corridor, baseline hearing, surgeon/center experience) and potential residual confounding. Selection into near-field monitoring based on feasibility and approach risks confounding by indication and non-exchangeability across cohorts; evolving electrodes, stimulus paradigms, and learning curves introduce secular trends that may bias modality contrasts.

Diagnostic studies exhibit heterogeneity of constructs (modality mix, threshold definitions, averaging/filters, and unstandardized response algorithms once thresholds were crossed). Many reports lack complete 2 × 2 tables, mix VS with other CPA lesions, or emphasize end-of-case status without intermediate windows; composite or shifting thresholds within cases further complicate harmonization. Small samples produce wide confidence intervals, and partial verification and spectrum effects may bias accuracy estimates; publication and language bias cannot be excluded.

Given k < 4 per construct and non-commensurate endpoints/time windows, we did not fit bivariate HSROC models and instead report study-level estimates to avoid pseudo-precision. This prioritizes internal validity but limits meta-analytic generalizability and precludes formal exploration of threshold effects or hierarchical covariance. Consequently, comparative signals (including the apparent advantage of CNAP) and construct-level diagnostic patterns should be considered hypothesis-generating and context-dependent, rather than definitive rankings of monitoring strategies.

### Future directions

Moving from prognostic association to stronger causal inference will require evaluation of standardized, auditable threshold sets, rather than further isolated single-center prognostic cohorts. This study and recent reports point to complementary operating characteristics—far-field ABR/BAEP trends are sensitive but less specific, whereas near-field channels deliver higher-SNR, faster updates whose sustained changes align more closely with outcome; moreover, standardized BAEP indices (e.g., STIAS–Am-V/La-V with contralateral referencing) materially improve discrimination and yield explicit cut-offs. A pragmatic multicenter design (cluster randomization, stepped-wedge, or target-trial emulation if randomization is infeasible) should test a prespecified bundle (e.g., ABR wave-V latency + 1.0–1.5 ms or amplitude − 50%; sustained Wave-V loss; CNAP/DCAP − 50%/−80% or loss) with time-stamped responses, enabling separation of diagnostic signal from treatment behavior and mitigating center/era effects. This agenda is supported by cohort evidence showing stage-linked correlations for ABR with TT-ECochG, prospective associations for CNAP presence/decrements with intelligible/serviceable hearing, and the performance gains reported for standardized BAEP metrics in short-term hearing preservation [12].

On the diagnostic track, once ≥ 4 commensurate datasets per construct are available, undertake bivariate HSROC—ideally IPD-based—to contrast trend-based vs. sustained-loss rules and far- vs. near-field modalities, harmonizing outcome windows (Immediate/Early/Long-term) and definitions (GR vs. AAO-HNS). Method-of-measurement development should prioritize atraumatic, self-retaining near-field electrodes, more robust DNAP arrays, and integrated multi-channel platforms (ABR + CNAP/DNAP ± TT-ECochG) with synchronized clocks to support fusion-based metrics; systems should undergo external validation before deployment. Finally, adopt core outcome sets with fixed windows and report complete 2 × 2 data where applicable to improve transportability and enable IPD synthesis—steps that directly address current heterogeneity, small-study imprecision, and incomplete reporting demonstrated in the existing literature and in our review.

## Conclusion

The available evidence supports the use of intraoperative auditory monitoring in hearing-preservation VS surgery and suggests a plausible incremental advantage for near-field techniques when feasible. Far-field ABR offers sensitive, widely deployable surveillance; near-field CNAP/DCAP provides faster, more specific adjudication of injurious events. However, multidimensional heterogeneity, nonrandomized designs, and incomplete reporting constrain the precision and transportability of effect estimates. Standardized, auditable threshold–action pathways and prospective, methodologically rigorous studies are the necessary next steps to translate promising physiology into reproducible gains in long-term, serviceable hearing.

## Supplementary Information

Below is the link to the electronic supplementary material.

Supplementary Material 1 (DOCX 969 KB)

Supplementary Material 2 (DOCX 269 KB)

## Data Availability

Data included in the study are available from the corresponding author upon reasonable request.
