# Supplementary material for: Intraoperative auditory monitoring in vestibular schwannoma surgery: Diagnostic accuracy and interventional effectiveness — a systematic review
Source: Neurosurg Rev. 2026 Apr 27;49(1):380. doi: 10.1007/s10143-026-04293-y (PMC13111521; doi:10.1007/s10143-026-04293-y)
Supplement: Supplementary file 1 — Supplementary Material 1 (DOCX 969 KB) [file 10143_2026_4293_MOESM1_ESM.docx]

**SAFE-adapted Stopping Rule and Outputs**

Title/abstract screening was performed in ASReview LAB (TF-IDF features, logistic regression, max query strategy). We initialized the model with 12 relevant sentinel records (ABR/BAEP, CNAP/DNAP, and hybrid ABR+near-field across comparative-effectiveness and diagnostic-threshold designs) and 12 clearly irrelevant controls, a balanced seed sufficient to stabilize early ranks without overweighting any modality or era. To balance high recall with efficiency, we applied a single, pre-specified composite stop requiring both a streak-based and a rate-based signal, followed by an external audit. Screening was provisionally halted only when (i) there were zero relevant hits in the last 100 screened records (zero-yield window) and (ii) the yield in the most recent 120 records was ≤1% (i.e., 0–1 relevant). These conditions address different failure modes: (i) detects an extended dry run; (ii) guards against local burstiness (thereby reducing premature stopping). Upon triggering (i)+(ii), we drew a simple-random audit of 15% of the unscreened tail (minimum 20 records). If the audit contained no additional relevant studies, we accepted the stop, invoking the conservative “rule of three” (3/n) to verify compatibility with a ≥95% recall target. If the audit identified any relevant study, screening resumed (continuing model-prioritized review of the tail) and termination was reconsidered only after both conditions were re-met and a repeat audit was clean. Retrieval of all sentinel records was confirmed prior to final termination. On completion, ASReview ranks, decisions, and settings were exported to preserve a full audit trail; purely technical/feasibility reports without clinical outcomes were retained narratively and excluded from quantitative synthesis.


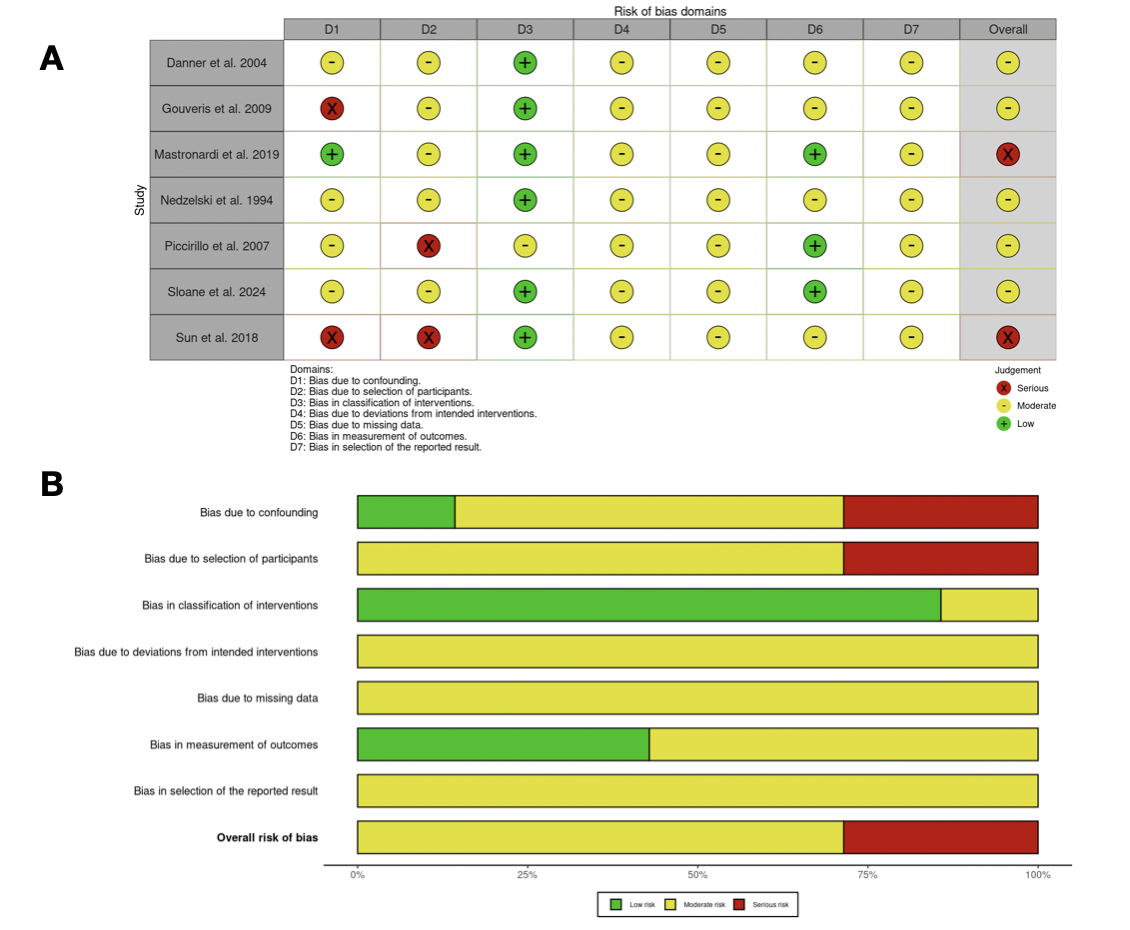


Figure Supp1: ROBINS


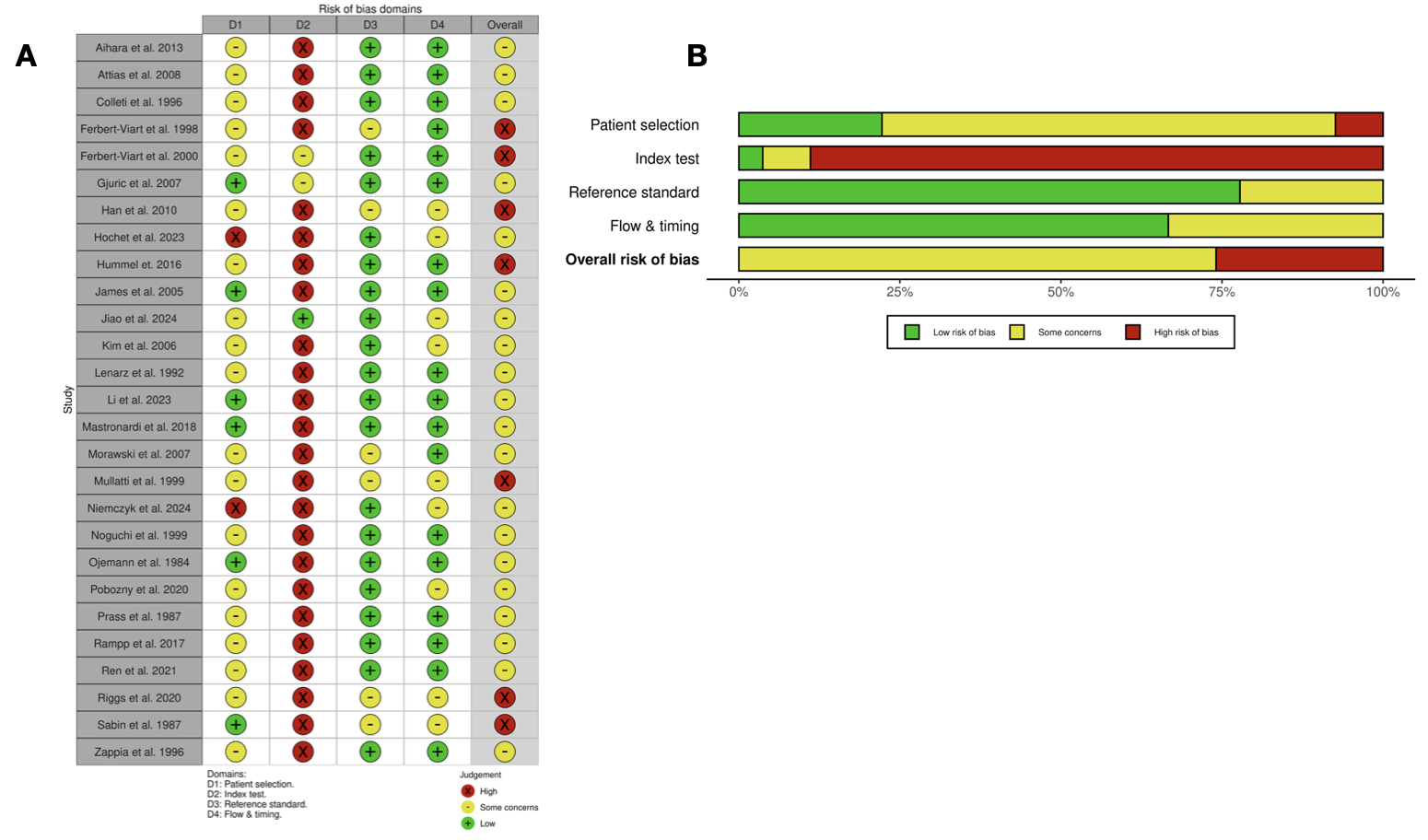


Figure Supp2: QUADAS-2
